# ^90^Y SPECT/CT quantitative study and comparison of uptake with pretreatment ^99 m^Tc‐MAA SPECT/CT in radiomicrosphere therapy

**DOI:** 10.1002/acm2.12512

**Published:** 2019-01-09

**Authors:** Senait Aknaw Debebe, Malek Adjouadi, Seza A. Gulec, Juan Franquiz, Anthony J. McGoron

**Affiliations:** ^1^ Department of Biomedical Engineering Florida International University Miami FL USA; ^2^ Department of Electrical and Computer Engineering Florida International University Miami FL USA; ^3^ Herbert Wertheim College of Medicine Florida International University Miami FL USA; ^4^ Radiological Physics of South Florida Miami FL USA

**Keywords:** ^90^Y, ^99m^Tc, dosimetry, radiomicrosphere therapy, SPECT/CT

## Abstract

**Introduction:**

Yttrium‐90 (^90^Y) microsphere post‐treatment imaging reflects the true distribution characteristics of microspheres in the tumor and liver compartments. However, due to its decay spectra profile lacking a pronounced photopeak, the bremsstrahlung imaging for ^90^Y has inherent limitations. The absorbed dose calculations for ^90^Y microspheres radiomicrosphere therapy (RMT) sustain a limitation due to the poor quality of ^90^Y imaging. The aim of this study was to develop quantitative methods to improve the post‐treatment ^90^Y bremsstrahlung single photon emission tomography (SPECT)/computed tomography (CT) image analysis for dosimetric purposes and to perform a quantitative comparison with the ^99m^Tc‐MAA SPECT/CT images, which is used for theranostics purposes for liver and tumor dosimetry.

**Methods:**

Pre and post‐treatment SPECT/CT data of patients who underwent RMT for primary or metastatic liver cancer were acquired. A Jasczak phantom with eight spherical inserts of various sizes was used to obtain optimal iteration number for the contrast recovery algorithm for improving ^90^Y bremsstrahlung SPECT/CT images. Comparison of uptake on ^99m^Tc‐MAA and ^90^Y microsphere SPECT/CT images was assessed using tumor to healthy liver ratios (TLRs). The voxel dosimetry technique was used to estimate absorbed doses. Absorbed doses within the tumor and healthy part of the liver were also investigated for correlation with administered activity.

**Results:**

Improvement in CNR and contrast recovery coefficients on patient and phantom ^90^Y bremsstrahlung SPECT/CT images respectively were achieved. The ^99m^Tc‐MAA and ^90^Y microspheres SPECT/CT images showed significant uptake correlation (*r* = 0.9, *P* = 0.05) with mean TLR of 9.4 ± 9.2 and 5.0 ± 2.2, respectively. The correlation between the administered activity and tumor absorbed dose was weak (*r* = 0.5, *P* > 0.05), however, healthy liver absorbed dose increased with administered activity (*r* = 0.8, *P* = 0.0).

**Conclusions:**

This study demonstrated correlation in mean TLR between ^99m^Tc‐MAA and ^90^Y microsphere SPECT/CT.

## 1 INTRODUCTION

Radiomicrosphere therapy (RMT) using Yttrium‐90 (^90^Y) microspheres is an effective liver‐directed therapy in the management of primary and metastatic liver cancer.^1^, ^2^, ^3^ The biodistribution of ^90^Y microspheres after treatment is assessed through ^90^Y bremsstrahlung imaging, preferably SPECT/CT.^4^, ^5^, ^6^ A single time point imaging is used due to the permanent implant of the microspheres where there is no elimination, redistribution and washout phases of the radiopharmaceutical.^7^

Technetium‐99m macroaggregated albumin (^99m^Tc‐MAA) scanning is performed prior to RMT as a surrogate for ^90^Y microspheres. In RMT planning, ^99m^Tc‐MAA planar and single photon emission tomography (SPECT)/computed tomography (CT) imaging is used to measure the percentage of particles that shunt to the lungs (lung shunt fraction, LSF), to assess any extrahepatic particle deposition, and to calculate the tumor to normal liver ratio (TLR).^1^, ^8^ Various authors have investigated the correlation of uptake and distribution between ^99m^Tc‐MAA and ^90^Y microspheres SPECT/CT.^8^, ^9^ The authors used tumor to normal liver ratio to evaluate the uptake on ^99m^Tc‐MAA and ^90^Y microsphere SPECT/CT images.

^90^Y microspheres bremsstrahlung SPECT/CT imaging could potentially identify an extrahepatic uptake. Early detection of such an uptake, thus, could initiate preventative measures early on. The tumor and liver dose estimates obtained from ^90^Y microspheres bremsstrahlung SPECT/CT imaging could be correlated with tumor response and liver toxicity clinical data.^10^ The major problem in ^90^Y bremsstrahlung imaging is the lack of a pronounced photopeak energy due to the continuous and broad energy spectrum of bremsstrahlung photons giving it a poor image quality. As a result, various studies based on phantom and Monte Carlo (MC) simulation have recommended appropriate energy windows in accordance with the collimator used.^11^, ^12^, ^13^, ^14^ It has been shown, often in conjunction with phantom studies that the incorporation of MC simulations to clinical images gives an optimal accuracy of bremsstrahlung images by compensating for photons through correction for attenuation, scatter, and collimator‐detector response.^13^, ^14^, ^15^ Alternately, studies have demonstrated the feasibility of ^90^Y PET/CT imaging excelling in contrast and resolution compared to bremsstrahlung SPECT/CT.^16^, ^17^ A recent study by Yue et al.^17^ concluded that both modalities are comparable for post‐RMT dosimetry estimation if appropriate reconstruction for ^90^Y bremsstrahlung SPECT/CT is applied. However, an appropriate reconstruction method requires incorporation of MC simulation in ^90^Y bremsstrahlung SPECT/CT for quantitative improvement, but is not easily applied for clinical implementation. Another recent study by Siman et al.^18^ developed a practical imaging protocol called background compensation for ^90^Y bremsstrahlung SPECT/CT imaging as an alternative to the MC method. The authors assert that their method doesn't address the main image degrading factors in ^90^Y bremsstrahlung SPECT/CT imaging, such as object scatter, septal penetration, and backscatter; nonetheless, they reported an improvement in recovery coefficient from 39% to 90% in a 37 mm sphere in a 10 mm volume of interest.

In this study, our aim was to apply post‐reconstruction techniques to improve the quantitative quality of ^90^Y bremsstrahlung SPECT/CT images and evaluate the correlation of the uptake pattern on the ^90^Y microspheres SPECT/CT post‐therapy images vs pretreatment ^99m^Tc‐MAA SPECT/CT images. We developed an iterative deconvolution based algorithm to improve the quantitative quality of ^90^Y bremsstrahlung SPECT images. This work provides a quantitative comparison of the uptake distribution in the ^99m^Tc‐MAA and ^90^Y microsphere SPECT images to increase the accuracy of the comparison. Tumor segmentation based on SPECT images is feasible in bremsstrahlung images since the deconvolution correction method results in images with more concentrated uptake in areas where the tumor is (excluding necrotic tumor) vs a dispersed or partially uniform distributions observed in the uncorrected 90Y bremsstrahlung SPECT images. To our knowledge, no work to date, has performed quantitative improvement on ^90^Y bremsstrahlung SPECT images prior to comparison with ^99m^Tc‐MAA SPECT/CT images. Furthermore, we analyzed the association between administered activity and liver dosimetry in patient study.

## 2 MATERIALS AND METHODS

### 2.A SPECT/CT image acquisition and reconstruction

All data were acquired on a dual‐head Philips Precedence 16P SPECT/CT (Philips Medical Systems Inc., USA).

#### 2.A.1 Patient study

Patient data were acquired as part of a retrospective study approved by the Florida International University Institutional Review Board. Therefore, written patient informed consent was not sought nor documented and image data were handled anonymously. In total, SPECT/CT data of 12 patients who underwent RMT with ^90^Y‐labelled resin microspheres (SIR‐spheres; Sirtex) were acquired. All patients had undergone ^99m^Tc‐MAA SPECT/CT scan prior to RMT. Mean administered ^99m^Tc‐MAA was 190 MBq. Data were collected for patients whose LSF ≤ 5% to minimize error due to extrahepatic deposition consistent with another group.^18^ The acquisition energy window was centered at 140 keV ± 15% for ^99m^Tc‐MAA SPECT with 128 × 128 projection matrix over 360°, for 20 s per azimuth on a low‐energy high‐resolution collimator. Image reconstruction was done using the ordered subset expectation maximization (OSEM) algorithm with three iterations and 16 subsets, 132 × 132 matrix and 4.664 × 4.664 × 4.664 mm^3^ voxel size. ^90^Y bremsstrahlung SPECT/CT was performed with a medium energy general purpose collimator with energy window centered on 90 keV ± 15%, 35 s per azimuth for 2 × 64 views over 360° (currently a standard protocol at our center). Image reconstruction for ^90^Y bremsstrahlung SPECT was performed using a built‐in three‐dimensional (3D) OSEM algorithm. ^90^Y bremsstrahlung SPECT images were reconstructed with four iterations and eight subsets, 132 × 132 matrix and 4.664 × 4.664 × 4.664 mm^3^ voxel size.

#### 2.A.2 Phantom study

The Jasczak phantom with eight fillable spherical inserts of inner diameter 2, 8, 10, 12, 16, 25, 31, and 34 mm was used. The spheres (multiple sizes, see Table 1) and background (~6 L volume) were filled with Yttrium‐90 (III) chloride solution (PerkinElmer Inc., Waltham, MA, USA) of activity concentrations 0.52 and 0.04 MBq/ml respectively, with an approximate sphere to background activity concentration ratio of 13:1. Total activity was 27 and 255 MBq in the spheres and background, respectively. Table 1 shows activities within the different sphere sizes. ^90^Y activity was diluted and measured in a 60 ml vial before adding to the spheres. The activity inside each sphere was measured using a Capintec dose calibrator (read out scale factor = 10). Imaging was set identical to patient studies in terms of imaging window, collimator, and image reconstruction.

**Table 1:** Activities with the hollow spheres

Sphere no	Sphere size in diameter (mm)	Activity (MBq)
S1	34	11.10
S2	31	8.88
S3	25	4.44
S4	16	1.11
S6	12	0.56
S7	10	0.28
S8	8	0.14
S9	2	~0

#### 2.A.3 Image processing

A MATLAB^®^ algorithm was developed to import and export images for Volumes of interest (VOIs) generation, semi‐automatic tumor segmentation, activity estimation, absorbed dose estimation, and statistical and mathematical analysis. For the patient studies, VOIs were drawn manually on the CT image slices. Binary masks from the CT images were mapped onto the respective SPECT scans. For the phantom study, eight circular VOIs were manually drawn on the CT slices, where knowledge of the phantom composition allowed us to identify the spheres and make sure the volumes in VOIs were consistent with the true measured sphere volumes. Background VOIs consisted of all voxels within the phantom boundary excluding voxels that belong to the spheres’ VOIs.

### 2.B Contrast recovery

We propose a new method based on an iterative expectation maximum likelihood deconvolution technique to improve the quantitative quality of the ^90^Y bremsstrahlung SPECT images. The Richardson–Lucy (RL) technique was used for post‐reconstruction image deconvolution implemented with MATLAB^®^. The method is an iterative expectation maximum likelihood deconvolution algorithm [Eq. (1)] where images degraded by the point spread function (PSF) of the detector and additive noises are corrected.^19^, ^20^ The choice of a maximum likelihood algorithm has the benefit of producing good quality images in the presence of high noise levels by preserving positive values through accounting for fluctuation in the signal and thus limiting noise amplification.^20^

(1) $$A_{i}^{\prime }=\left\{\left[\frac{A}{A_{i-1}^{\prime }⊗PSF}\right]⊗PSF\right\}A_{i-1}^{\prime }$$

*A* is the reconstructed image degraded by the response of the detector, *A’* is the contrast recovered image where $A_{0}^{\prime }$ = *A*, ⊗ is the 3D (x, y, z) convolution operation and *i* is the iteration number. In our study the *PSF* was fixed and the only iterative maximum likelihood estimate was the image. The PSF of the collimator‐detector response was modeled by the Gaussian function according to Eq. (2) where *σ* was found from the relationship with full‐width at half‐maximum (FWHM) of the detector, $\text{FWHM}=\sqrt{8\ln(2)}$ × σ.

(2) $$PSF\left(x,y,z\right)=\frac{1}{{\left(2\pi \right)}^{\frac{3}{2}}\sigma^{3}}e^{-\left(x^{2}+y^{2}+z^{2}\right)/2\sigma^{2}}$$

Improvements in the quantitative quality ^90^Y bremsstrahlung SPECT/CT images were evaluated using contrast to noise ratio (CNR) and contrast recovery coefficients (Q_H_)^16^ for the patient and phantom studies respectively as given by Eqs. (3)) and (4). Our analysis of quantitative image improvement is based on matched VOIs between the CT and SPECT images as this is required for dosimetry estimation.

(3) $$CNR=\frac{M_{T}-M_{B}}{\sqrt{M_{B}}}$$

(4) $$Q_{H}=\frac{C_{S}/C_{B}^{-1}}{R-1}\times 100$$

**Figure 1:**
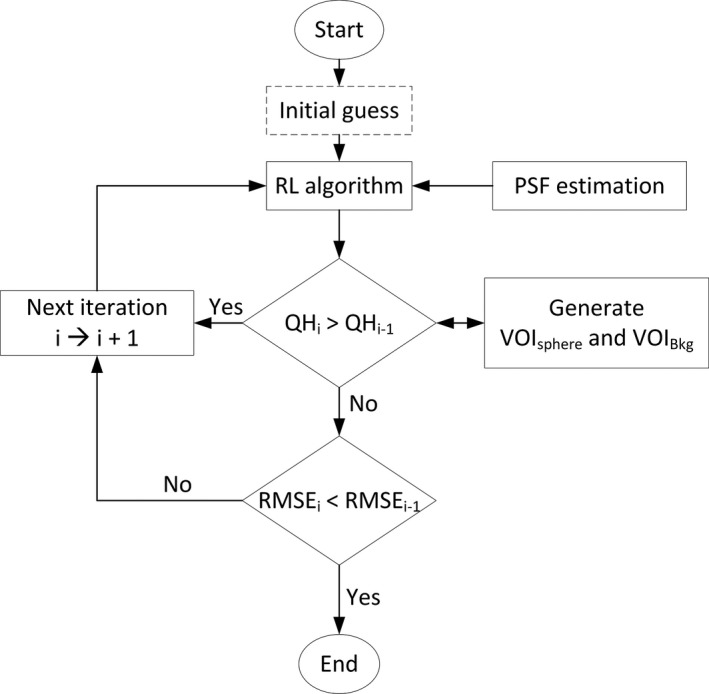
Block diagram of the Richardson–Lucy algorithm employed.

*M*_*T*_ is the mean count in tumor VOIs, *M*_*B*_ is the mean count in healthy liver VOIs, *C*_*S*_ is the mean count in the sphere VOIs, *C*_*B*_ is the mean count in the background VOIs and *R* is the true sphere to background ratio. The block diagram of the employed RL algorithm is shown in Fig. 1.

**Figure 2:**
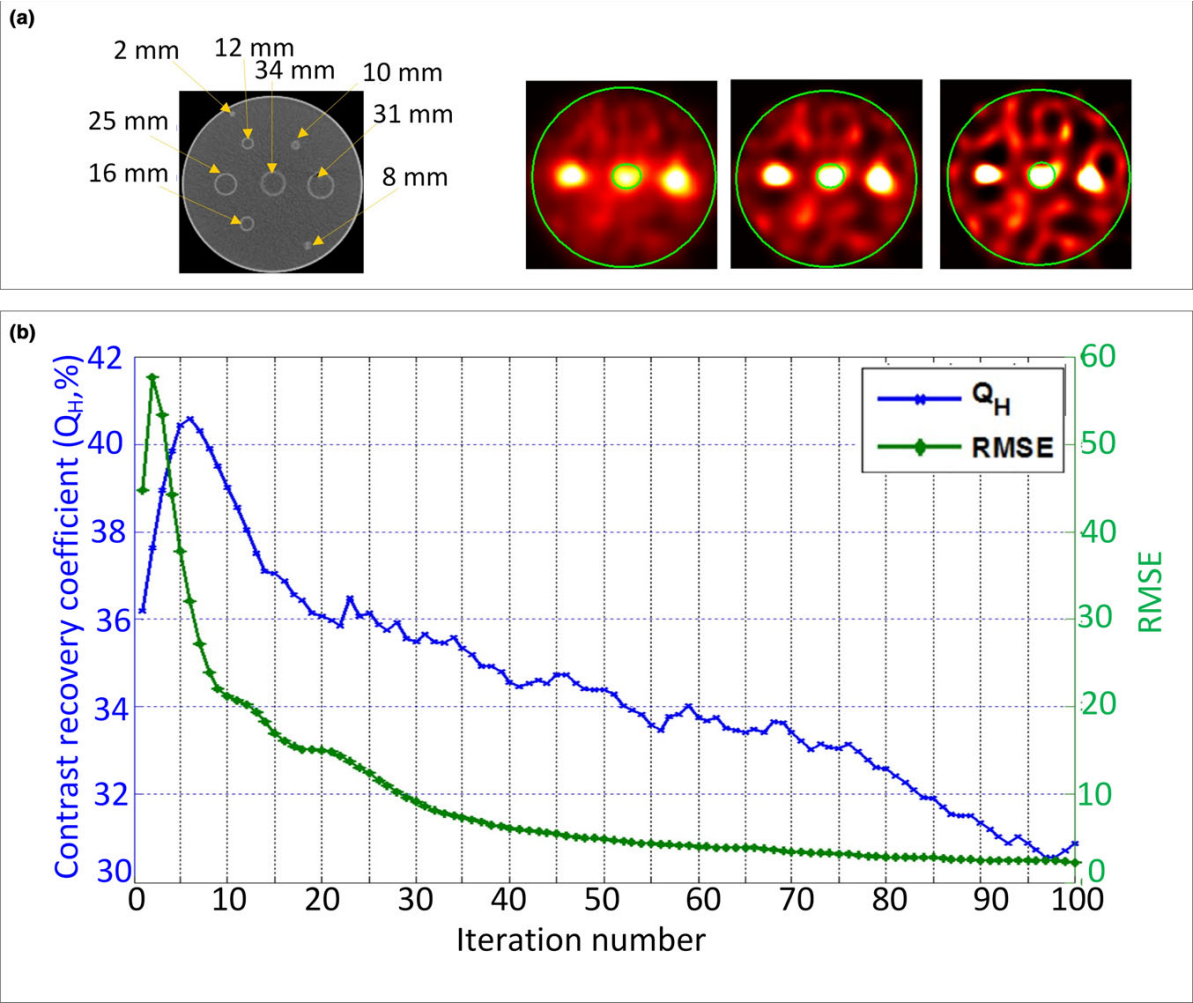
(a) Phantom computed tomography (CT) scan and single photon emission tomography (SPECT) images for iteration numbers of 0, 6, and 15 left to right. (b) Plot of Q_H_ (right axis) and root mean square error (RMSE) (left axis) vs iteration number for the 34 mm sphere

The iteration number for the algorithm was chosen at the point of maximum likelihood where the contrast recovery coefficient for the 34 mm sphere was at its maximum value, which corresponded to a decline in the associated root mean square error (RMSE) between two consecutive iterative image estimates, which was found to be at the sixth iteration as shown in Fig. 2. The contrast improvement algorithm using Richardson‐Lucy deconvolution is a new idea integrated in this approach.

### 2.C SPECT calibration factor measurement

The calibration factor (CF) was defined as the ratio of the total reconstructed counts to the true activity. Due to the continuous nature of the bremsstrahlung x‐ray spectrum that results in the absence of a photopeak window, a CF calculated using a point source might not quite represent photon counts in the clinical setup. As a result, mostly phantom studies are used for estimating a CF in ^90^Y bremsstrahlung SPECT/CT imaging. We used two methods for calculating the CF from the ^90^Y bremsstrahlung SPECT/CT images of the 12 patients. The first approach (method 1) used the entire total reconstructed counts within the field of view (FOV) of the SPECT detector. The second approach (method 2) used counts from the liver segments only. In method 2 the liver was first segmented as described in the image processing section and total counts within the liver VOIs were taken. This method avoids counts due to image artifacts. The motivation for using directly the patients’ liver uptake to calculate the CF is that due to the proximity of the treatment to the target area where the ^90^Y microspheres are deposited predominantly in the liver, the ^90^Y activity can be determined with high accuracy.^21^ Calibration curves were generated using linear regression analysis to derive the relationship between patient administered activity and reconstructed counts. The CFs were calculated from the slopes of the calibration curves.

For the phantom study, the total reconstructed counts within the phantom boundary were considered. The total activity was the sum of the activities inside the spheres and the background. The CF from the phantom study was evaluated to be within the 95% confidence interval (CI) of the calibration curves from the ^90^Y microsphere patient studies to validate its application on the phantom images.

### 2.D Activity estimation and comparison with the administered dose

The ^90^Y bremsstrahlung SPECT reconstructed pixel values were divided by the CF to convert values in counts per second (cps) into units of activity (MBq). For the patient studies, total liver activity estimation was compared to the administered activity. Activities inside the eight spherical inserts were compared to the activities measured using the dose calibrator for the phantom study. Percent error was calculated as % Error = (True Activity − Estimated Activity)/True Activity × 100%, where true activity is the administered activity. To compare the correlation between uptake distribution on ^99m^Tc‐MAA and ^90^Y microsphere SPECT/CT images, the two images were co‐registered.

### 2.E Co‐registration of ^99m^Tc‐MAA and ^90^Y microspheres SPECT/CT

Since the SPECT images were separately registered to the CT images we tried three approaches. The first method was taking single CT images as a reference (“fixed image”) and the SPECT images as a source (“moving image”). The second method is CT‐to‐CT registration. In the third method, ^90^Y microsphere SPECT was taken as the reference image and the ^99m^Tc‐MAA SPECT was the source image (SPECT‐to‐SPECT). Two image registration tools were used, FMRIB's Software Library (FSL) and Statistical Parametric Mapping (SPM), both well‐established tools extensively used for registering anatomical and functional images, and we found visually the best results using SPM.^22^ We used the spatial normalization function of SPM12 using the standard settings of the toolbox. In order to choose the best registration approach, we calculated the mutual information between the co‐registered ^90^Y microsphere and ^99m^Tc‐MAA SPECT images from the methods. The mutual information between the two images is defined as:

(5) I(A,B)=H(A)+H(B)−H(A,B)

*H (A)* and *H (B)* are the entropy of image *A* and *B,* respectively, and *H (A, B)* is their joint entropy. The method which gave the highest *I (A, B)* was used for registering the images. Details of the calculation of mutual information is explained by Maes et al.^23^ Mean CPU time to register two images was about 15 min on a standard PC.

### 2.F Tumor segmentation and tumor to liver ratio calculation (TLR)

Quantitative uptake analysis on ^99m^Tc‐MAA SPECT/CT for tumor delineation is usually done using an isocontour method with a dedicated software.^8^ In our study tumor segmentation based on the uptake of ^99m^Tc‐MAA and ^90^Y microspheres was performed with an active contour segmentation method using the well‐known Chan‐Vese energy paradigm^24^ to delineate areas of high activity (tumor) from the surrounding low activity (healthy) regions. This method doesn't use a single threshold, which is an advantage in this case since the administered activities of ^99m^Tc‐MAA and ^90^Y microspheres are quite different. The method uses gradient based information between neighboring voxels in order to segment the higher uptake regions. The global gradient calculation is specific to the image, that is, the ^99m^Tc‐MAA and ^90^Y microspheres SPECT images are treated separately but in the same slice range. Due to the proximity of the treatment, higher regions of uptake are taken as tumor. The segmented tumor was subtracted from the whole liver volume to get the healthy part. Since counts are proportional to activity concentration, the TLRs were calculated by dividing the mean counts per pixel of the tumor by the mean counts per pixel of the healthy liver.

### 2.G Absorbed dose estimation

The voxel S‐value method was used to estimate 3D radiation absorbed dose in ^90^Y bremsstrahlung SPECT/CT images.^25^, ^26^ Cubical voxel S values for the pixel size of 4.664 mm were determined using linear interpolation from 3 and 6 mm pixel sizes calculated by Franquiz et al. and Bolch et al.^25^, ^26^ It is expected that errors are associated with using a linear interpolation for estimating S‐values for a specific cubic voxel size from tabulated results.^27^ For our retrospective study this error is acceptable as explained in MIRD pamphlet no 17.^26^ Absorbed dose per voxel was estimated by convolution of the activity images with the corresponding voxel S values [Eq. (5)], based on the MIRD Pamphlet No. 17.^26^

(6) $$D_{T}=A⊗S=A_{S1}\times S_{T\leftarrow S1}+A_{S2}\times S_{T\leftarrow S2}+A_{S3}\times S_{T\leftarrow S3}\ldots$$

D_T_ is the absorbed dose at the target voxel (mGy), A (MBq) is the cumulated activity from the surrounding source voxels, ⊗ is the 3D convolution and S is the voxel S‐value (mGy/MBq) for each associated source distance to the target voxel. Cumulative dose‐volume histograms (cDVHs) and isodose curves were generated for the tumor and healthy liver VOIs from the SPECT dose map images.

### 2.H Statistical analysis

Quantitative parameters are presented as mean ± SD and ranges. Linear regressions were generated between administered activities (independent variable) and cps for the purpose of predicting CFs. We report slope, *R* ^2^, standard error and 95% CI of the regression models. Pearson correlation coefficient and its *P* value were used to test for significance of correlations between TLRs from ^99m^Tc‐MAA and ^90^Y microsphere SPECT/CT as well as between administered activity and absorbed doses. Statistical analyses were deemed significant as having a *P *< 0.05. All statistical analyses were performed with Minitab^®^ software package (version 17).

## 3 RESULTS

### 3.A Contrast recovery

**Figure 3:**
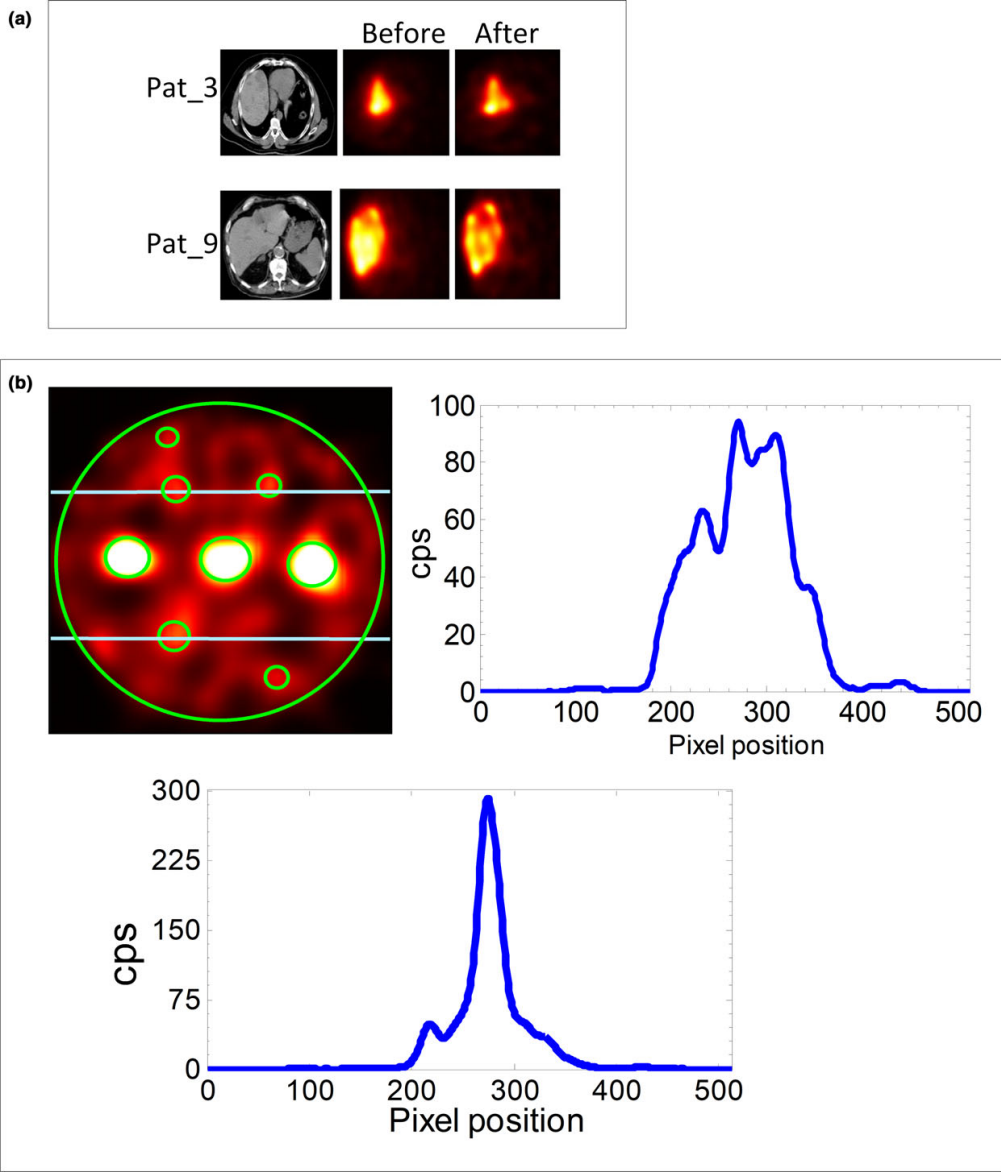
(a) Result of contrast recovery showing the before and after ^90^Y bremsstrahlung single photon emission tomography (SPECT) images of patients with the respective computed tomography (CT) scans. (b) Line profiles through the 16 mm (lower) and 12 mm (upper right) spheres of the phantom ^90^Y bremsstrahlung SPECT/CT image. Phantom SPECT images were resized to 512 × 512 for display purpose hence higher pixel position for the line profiles

Figure 3(a) shows examples of improvements in the CNRs between tumor and the background for patient ^90^Y bremsstrahlung SPECT/CT images. For the patients shown (Pat 3 and 9), the CNRs were respectively 18.9 and 48.8 before and 24.1 and 51.7 after contrast recovery. For the phantom study improvement in Q_H_ ranged between −8.3 to 41.0%. For the smallest spheres (2, 8, and 10 mm), no improvement in Q_H_ was found. Figure 3(b) shows line profiles along the 16 and 12 mm spheres. From the profiles, it can be seen that the signal in the 16 mm sphere significantly differs from the background while the signal within the 12 mm sphere and the background are indistinguishable.

### 3.B SPECT CF

Results of the regression analyses of the relationships between cps and administered activity gave the following results, the slopes being the CFs (cps/MBq); method 1, CF_1_ = 19 990 cps/MBq with 95% CI 14 081 ≤ *β *≤ 25 899 (*R* ^2^ = 0.85, standard error (SE) = 2652) and method 2, CF_2_ = 8316 cps/MBq with 95% CI of 6810 ≤ *β *≤ 9822 (*R* ^2^ = 0.94, SE = 676). The CF from the phantom study was 9049 cps/MBq, which falls within the 95% CI of CF_2_.

### 3.C Activity estimation and comparison with the administered dose

The total activity inside the liver was estimated for each patient using the two CFs. For CF_1,_ total liver activity estimation resulted in mean percent error of 59 ± 5%. Applying CF_2_ gave the smallest error (−5 ± 13%), thus it was used for subsequent analysis. Table 2 shows results of total activity estimation within the liver VOIs using CF_2_. For most of the patients the estimated total liver activity percent errors were within ±10% giving an overall satisfactory results. As our study is retrospective based on anonymized data, we couldn't provide possible clinical reasons for the larger deviations of the estimated activities in some of the patients. Results of activity estimates inside the spheres and the background for the phantom study gave total mean percent error of −23 ± 41%.

**Table 2:** Administered activities and total estimated activities inside liver VOI

Patient no.	Administered activity (MBq)	Estimated activity (MBq)	Error (%)
Pat_1	555	480	14
Pat_2	570	652	−14
Pat_3	759	967	−27
Pat_4	781	788	−1
Pat_5	966	1251	−30
Pat_6	999	1009	−1
Pat_7	1203	1202	0
Pat_8	1236	1221	1
Pat_9	1262	1288	−2
Pat_10	1436	1350	6
Pat_11	1517	1572	−4
Pat_12	2072	2188	−6
Mean	1113	1164	−5
SD	438	448	13

### 3.D Co‐registration of ^99m^Tc‐MAA and 90Y microspheres SPECT/CT

**Figure 4:**
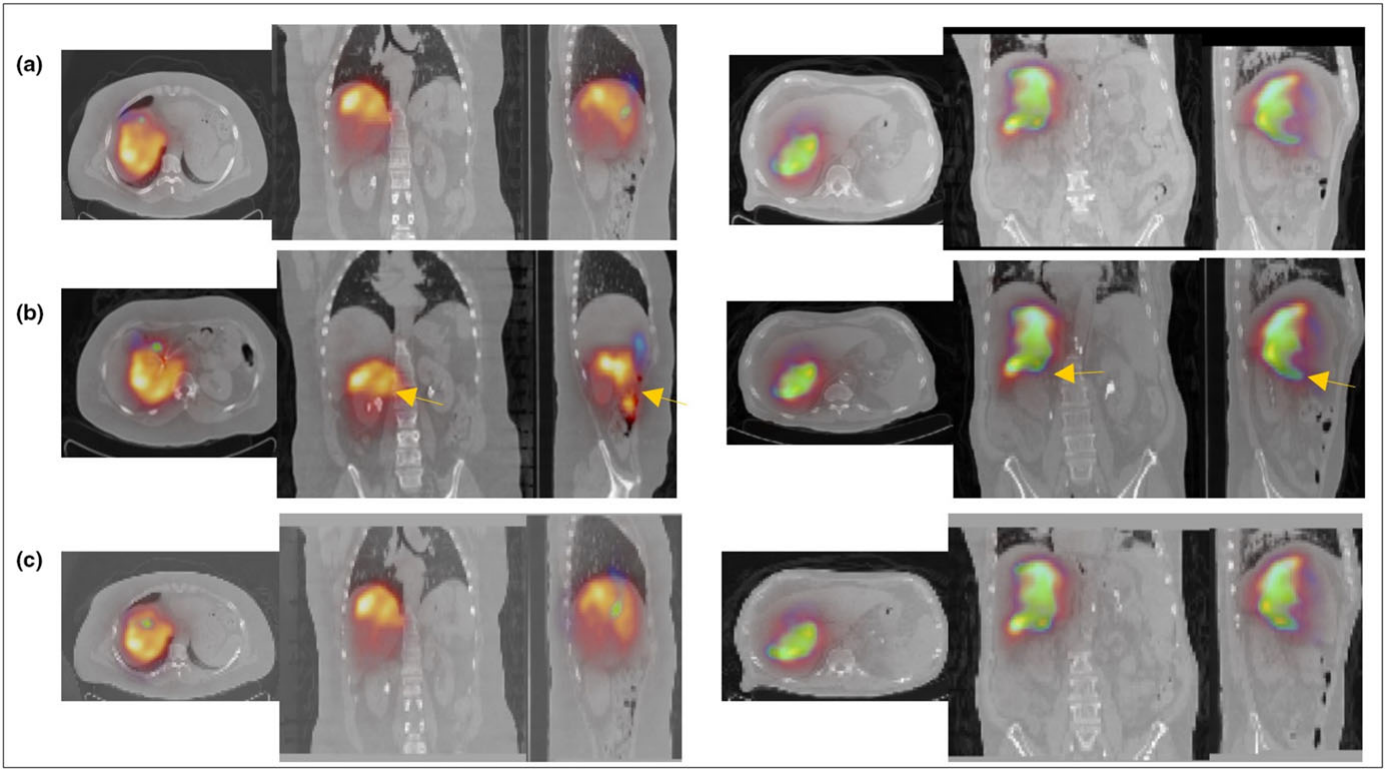
Patient 6 (left) and 8 (right) co‐registered computed tomography (CT) and single photon emission tomography (SPECT) images of 99mTc‐MAA (blue) and 90Y microsphere (red) for reference images of CT of post‐treatment (a), CT‐CT registration (b) and 90Y SPECT (c). The arrows in (b) show the misalignment between the CT and SPECT images showing activity distributions outside of the liver boundary in the coronal and sagittal views.

**Figure 5:**
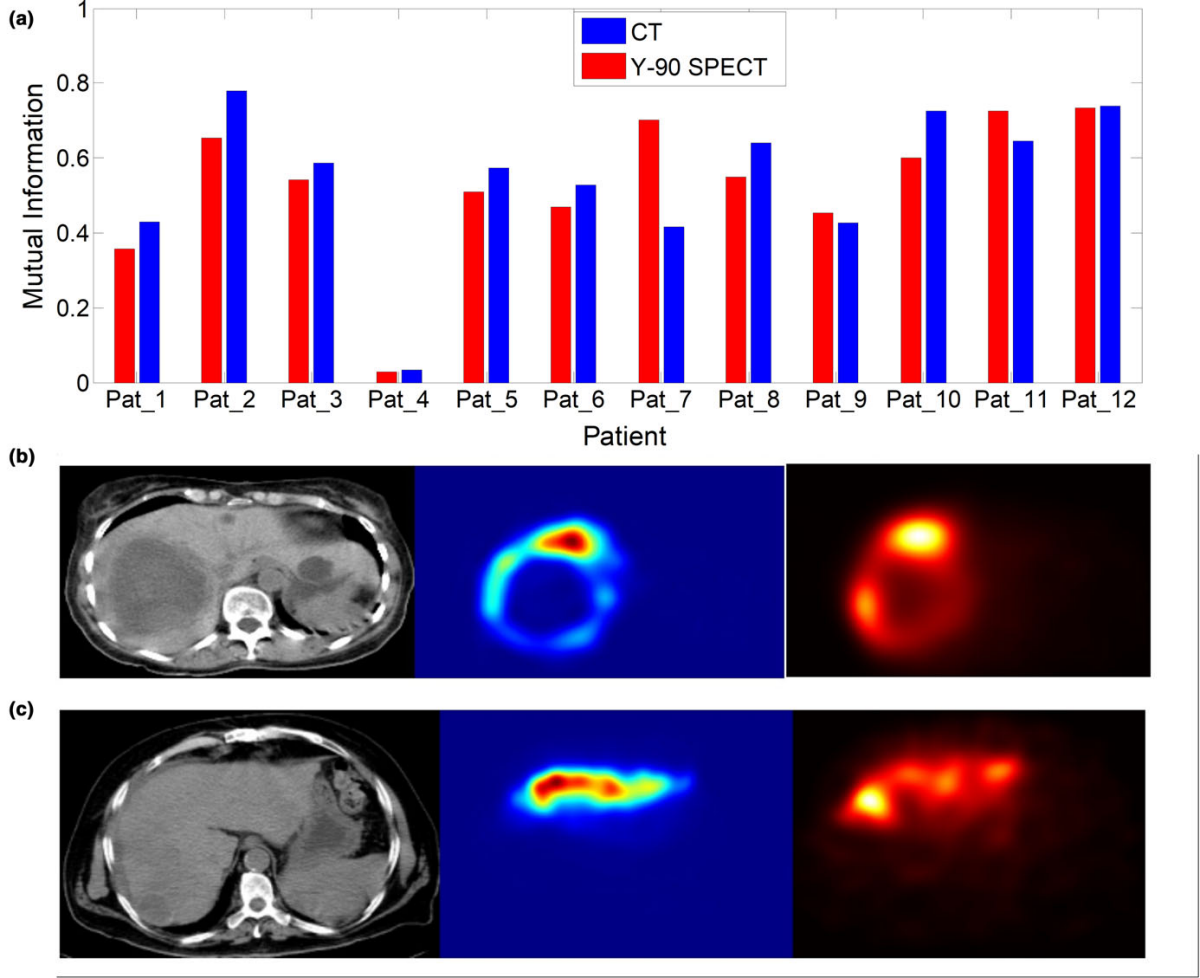
(a) Mutual information between co‐registered 90Y microsphere and 99mTc‐MAA SPECT images. (b) Patient 7 computed tomography (CT) (left), 99mTc‐MAA (middle) and 90Y microsphere (right) SPECT scans showing a minimal uptake within the liver. (c) Patient 4 CT (left), 99mTc‐MAA (middle) and 90Y microsphere (right) SPECT that showed the smallest mutual information between the co‐registered pre and post‐treatment images.

Figure 4 shows results of the three co‐registration approaches where the CT scan of the post‐treatment as a reference image gave the best alignment between the images. Using the ^90^Y microsphere SPECT image as a reference also gave a good result visually comparable to the aforementioned result [Fig. 4(c)]. But the mutual information is higher or equal in most patients where the CT scan was the reference image [Fig. 5(a)]. In other patients, for example, patient 7, the ^99m^Tc‐MAA and ^90^Y microspheres SPECT images didn't co‐register correctly with the CT image. From the SPECT scans of this patient, we observed that the patient had a hepatic tumor with a necrotic core with minimal uptake inside the liver which reduced the mutual information required for co‐registering the SPECT and CT scans [Fig. 5(b)]. Patient 4 showed the smallest of all mutual information between ^99m^Tc‐MAA and ^90^Y microsphere SPECT scans and similar to the previously mentioned patient, the SPECT scan didn't co‐register well with the CT scan. We observed that the ^99m^Tc‐MAA and ^90^Y microsphere SPECT scans of this patient have exclusively localized activity in the left lobe. For these patients image analyses on the ^99m^Tc‐MAA and ^90^Y microsphere SPECT/CT were performed separately.

### 3.E Tumor segmentation and TLR comparison

**Figure 6:**
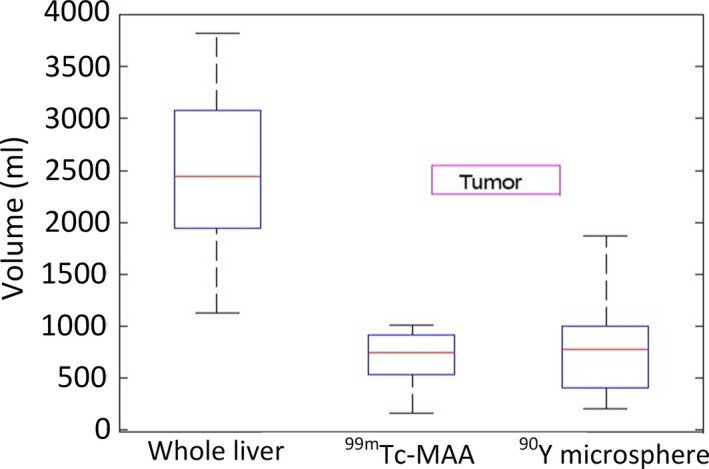
Box plot of whole liver and segmented volumes tumor.

For ^99m^Tc‐MAA, tumor volumes ranged between 160 and 1010 (695 ± 275) ml. ^90^Y‐microshpere uptake gave tumor volumes in the range of 207–1868 (786 ± 462) ml. Figure 6 shows the Box‐Whisker plot of the volumes including whole liver. ^90^Y‐microsphere SPECT/CT images gave an overall higher tumor volume for most patients compared to the ^99m^Tc‐MAA SPECT/CT images. A paired *t*‐test of the two volumes gave insignificant difference (*t* = −0.82, *P* = 0.429; Table 3).

**Table 3:** Result of paired *t*‐test between pre and post‐treatment tumor volumes

	N	Mean	SD	SE mean
Paired *T* for 99mTc‐MAA vs Y–90 microsphere				
99mTc‐MAA	12	695	275	79
Y–90 microsphere	12	786	462	133
Difference	12	−91	383	110

*T*‐test of mean difference = 0 (vs ≠ 0): *T* = −0.82.

*P* = 0.429.

**Figure 7:**
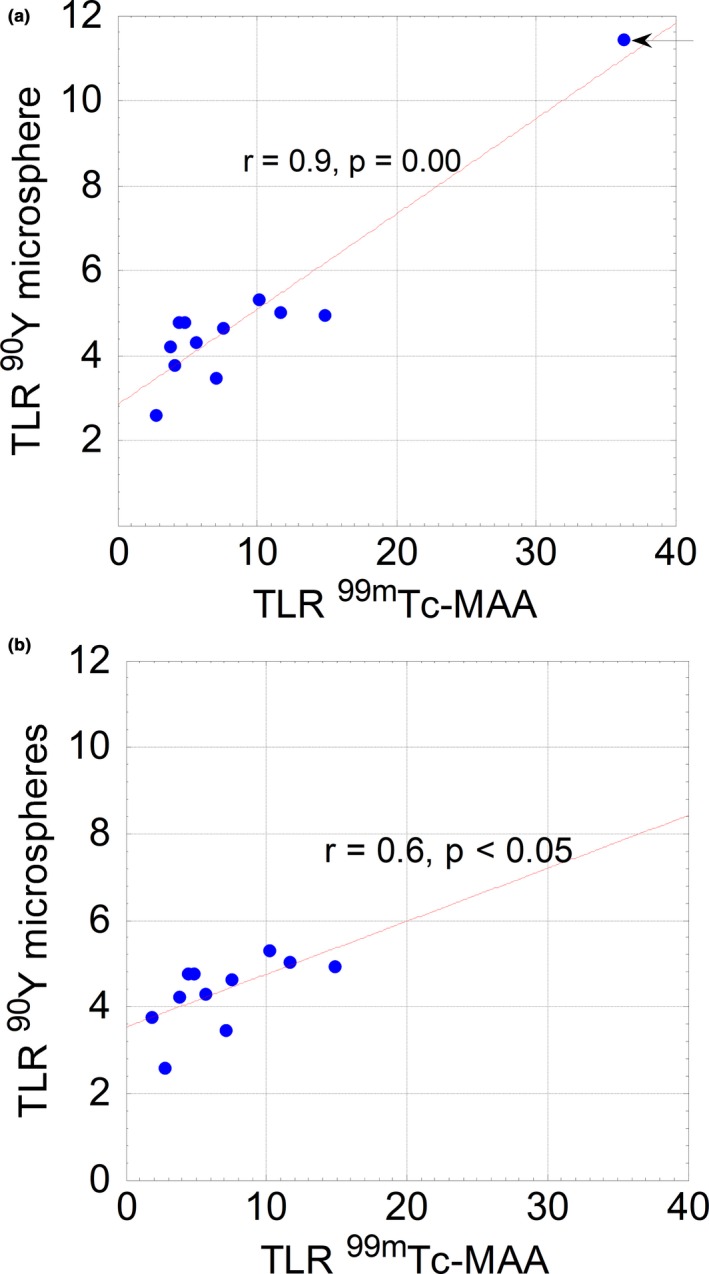
(a) Scatterplot illustrating correlation between mean tumor to healthy liver ratios in ^90^Y microspheres and ^99m^Tc‐MAA SPECT arrow indicating an outlier. (b) Outlier in (A) removed showing a reduced correlation between ^90^Y microsphere and ^99m^Tc‐MAA SPECT TLRs.

The total mean TLR was 9.4 ± 9.2 and 5.0 ± 2.2 on ^99m^Tc‐MAA and ^90^Y microsphere SPECT/CT, respectively. Figure 7 shows the scatterplot of mean TLRs from the two images displaying a significant correlation (*r* = 0.9, *P* = 0.00). From the plot, one patient appears to be an outlier (Grubbs’ outlier test, *P* = 0.00), and taking this patient out of the analysis gave a reduced correlation (*r* = 0.6, *P* < 0.05).

### 3.F Absorbed dose estimation

**Figure 8:**
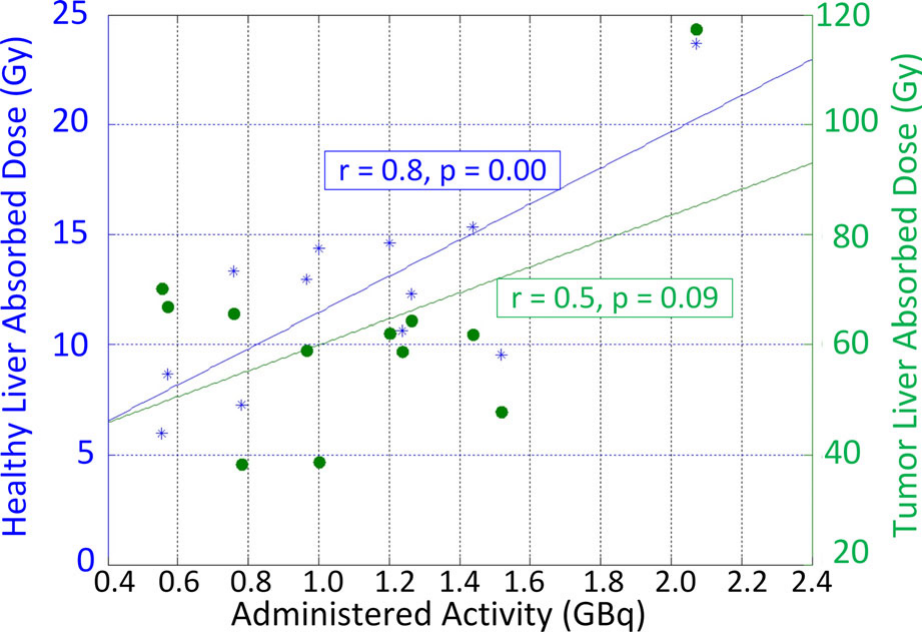
Correlation of administered activity with healthy liver and tumor absorbed doses.

**Figure 9:**
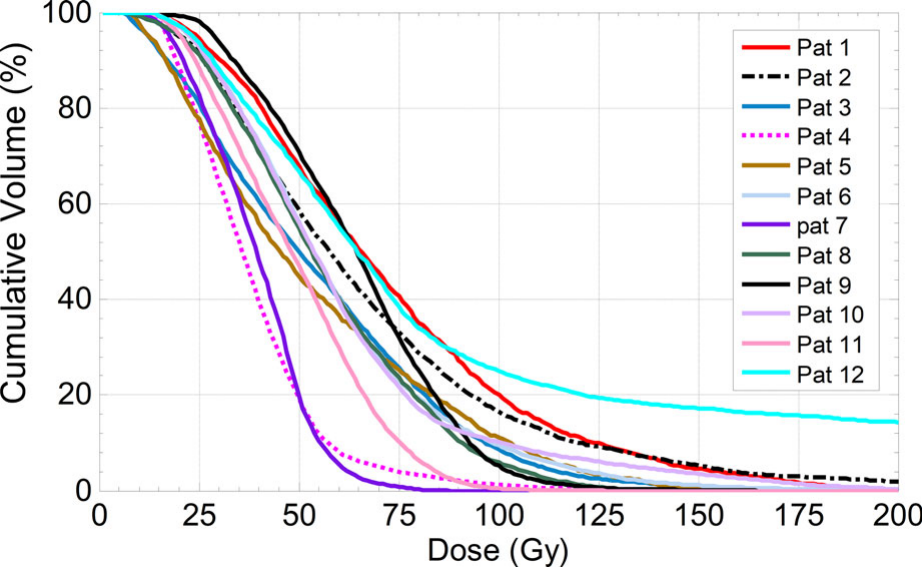
cDVH of tumor volumes for all patients.

**Figure 10:**
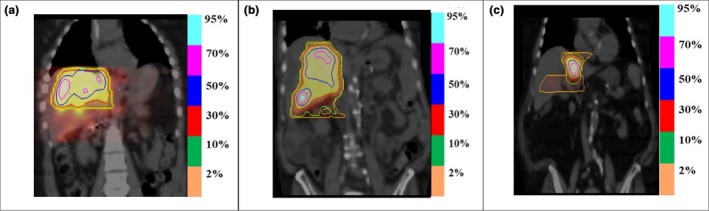
Coronal slice through single photon emission tomography (SPECT) dose map with isodose lines fused with computed tomography showing ^90^Y microsphere dose distribution for patients 6(a), 8(b), and 12(c).

The mean absorbed doses for the tumor and healthy liver from ^90^Y bremsstrahlung SPECT/CT images were 62.6 ± 20.2 (range: 38.4 to 117.2 Gy) and 12.4 ± 4.7 (range: 6 to 23.7 Gy), respectively. Figure 8 demonstrates the relationship between the administered activities and absorbed doses. Tumor absorbed dose showed non‐significant correlation with the administered activity (*r* = 0.5, *P* > 0.05) however, there was a strong correlation between healthy liver absorbed dose and the administered activity (*r* = 0.8, *P* = 0.00). Figure 9 shows the cDVH of ^90^Y microsphere uptake in the tumor segments for all the patients. Figure 10(a) shows dose distribution of ^90^Y microsphere SPECT/CT for patient 6 with pre and post‐treatment TLR of 2.7 and 2.6, respectively, Fig. 10(b) shows patient 8 with pre and post‐treatment TLR of 10.2 and 5.3, respectively and Fig. 10(c) shows dose distribution for patient 12 with pre and post‐treatment TLR of 14.7 and 5.0, respectively. The isodose lines represent percent of maximum absorbed dose uptakes.

## 4 DISCUSSION

The primary objective of this study was to develop a post‐reconstruction algorithm to quantitatively improve the ^90^Y bremsstrahlung SPECT/CT images. The second objective was to compare the uptake distribution of pretreatment ^99m^Tc‐MAA SPECT/CT and ^90^Y microspheres SPECT/CT images. In retrospect, the contribution of this study is in developing a clinically applicable method to improve the quantitative quality of ^90^Y bremsstrahlung SPECT/CT images for a more accurate quantitative comparison with ^99m^Tc‐MAA SPECT/CT images. The proposed algorithm for ^90^Y bremsstrahlung SPECT/CT image improvement uses the Richardson–Lucy technique for contrast recovery. The challenges for our method were image degradation due to object scatter, septal penetration, and backscatter. Despite the inherent shortcomings, we achieved a meaningful improvement in CNR and Q_H_ which were measured in clinical and phantom ^90^Y bremsstrahlung SPECT/CT images, respectively. The small sphere volumes (2, 8, and 10 mm) showed indifferent results before and after contrast recovery. The challenge to draw the ROIs introduces extra errors in addition to the fact that the volumes might be highly influenced by noise. The smallest volume that gave an acceptable result was the 16 mm sphere with substantial signal difference from the background. Our result of the highest Q_H_ for the 34 mm was ~41% using a matching VOI. Applying similar methods used by other authors to evaluate Q_H,_ 16, 28 that is using an ROI in the slice through the center of the spheres, we found a higher value of Q_H_ = 80% for the 34 mm sphere. For a true dose assessment, however, this calculation should base on equal sized VOIs between the CT and SPECT image of the sphere.^18^ In addition, using an activity similar to clinically administered ^90^Y microsphere is beneficial in increasing accuracy of the calculation due to an increase in sample size of the counting statistics.

With the clinically available reconstruction method and application of contrast recovery algorithm, total liver activity estimation gave percent error of −5 ± 13 using the CF found from counts within the liver VOIs. Siman et al.^18^ generated a global CF derived from patient studies where entire counts within the FOV were considered. The global CF was applied to an IEC phantom with a 37 mm sphere insert and the authors reported an error of −25% with respect to the true activity. For our phantom study, we used CF estimated from the total reconstructed counts, which was within 95% CI of the patient calibration curve, and obtained total mean percent error of −23 ± 41%. Sphere volumes ≤12 mm resulted in the highest error. Further research is required for clinical application in patients with high LSF values.

The predictive accuracy of ^99m^Tc‐MAA regarding the actual ^90^Y microspheres dose distribution and its impact on patient outcomes is a source of controversy. Ilhan et al.^9^ have studied the relationship between ^99m^Tc‐MAA and ^90^Y microspheres uptake in a retrospective study which involved 502 patients of various liver cancer types. This study found a weak correlation between the mean tumor to background (healthy liver) ratios of ^99m^Tc‐MAA SPECT/CT and ^90^Y microsphere SPECT/CT images. We believe a comparison of the pre and post‐treatment images of ^99m^Tc‐MAA and ^90^Y microspheres may help evaluate the discordance between MAA distribution vs actual microsphere distribution. There are known factors that contribute to discrepancies. First and foremost is the variable size and shape of the albumin particles and clusters. Additional factors may include the embolizing effect of ^90^Y microspheres and differences in positioning of the catheter tip between the ^99m^Tc‐MAA and ^90^Y microsphere procedures.

Prior to quantitative comparison, the pre and post‐treatment SPECT/CT images were co‐registered. The accuracy of the comparison strongly depends on the proper matching of the two images in all three dimensions. To minimize outlier effects, we used the ratio of the mean activities between the tumorous and healthy livers to calculate the TLRs. The mean TLRs between ^99m^Tc‐MAA and ^90^Y microsphere SPECT/CT images showed a significant correlation with one patient as an outlier. Ilhan et al.^9^ state that the TLRs from ^99m^Tc‐MAA SPECT/CT is higher in all patients compared to the respective ^90^Y microsphere SPECT/CT scans, which the authors believe to be due to the poor image quality of ^90^Y bremsstrahlung SPECT/CT. In our study, we found that after ^90^Y bremsstrahlung SPECT/CT image improvement the TRLs were higher in ^99m^Tc‐MAA SPECT/CT for most of the patients. The standard deviation of the TLRs on ^90^Y microspheres SPECT/CT images is lower than ^99m^Tc‐MAA SPECT/CT which could be associated with the embolizing effect of ^90^Y microsphere particles.^8^, ^9^

Dosimetry of ^90^Y microsphere distribution has the benefit of providing the real dose‐response relationship for further treatment if required, instead of the predicted dose‐response using ^99m^Tc‐MAA. From our dosimetry analysis, the administered activity showed a strong correlation with the absorbed dose in the healthy liver, however, the relationship was weak with tumor absorbed dose. There are several factors that could contribute to the correlation between the absorbed doses and the administered activity. The higher non‐uniform microsphere distribution within the tumor vasculature compared to the healthy liver could contribute to the more proportional dose deposition in the healthy liver parenchyma vs to the tumor region.^3^

## 5 CONCLUSION

In this study, we proposed a technique for quantitative image improvement of ^90^Y bremsstrahlung SPECT/CT with the objective of quantitative comparison with ^99m^Tc‐MAA SPECT/CT images and estimation of liver dosimetry. Within the limitation of our image correction method, the results of this study showed meaningful quantitative improvement of ^90^Y bremsstrahlung SPECT/CT images in phantom and patient studies. We identified a correlation in mean TLRs between ^99m^Tc‐MAA and ^90^Y microspheres SPECT/CT uptake distribution. In addition, a linear relationship was observed between administered activity and absorbed dose in the healthy liver while the relation was insignificant with tumor absorbed dose.

## CONFLICT OF INTEREST

Adjouadi M. received research grant from National Science Foundation (CNS‐1532061). The other authors declare no conflicts of interest.

## ETHICAL APPROVAL

This retrospective study was performed in accordance with the 1964 Declaration of Helsinki and was approved by the Institutional Review Board of Florida International University (Number: 104882). Informed consent was waived for all patients included in this retrospective study.
